# Computerized tomography texture analysis of pheochromocytoma: relationship with hormonal and histopathological data

**DOI:** 10.1007/s40618-022-01826-2

**Published:** 2022-06-10

**Authors:** A. De Leo, G. Vara, A. Paccapelo, C. Balacchi, V. Vicennati, L. Tucci, U. Pagotto, S. Selva, C. Ricci, L. Alberici, F. Minni, C. Nanni, F. Ambrosi, D. Santini, R. Golfieri, G. Di Dalmazi, C. Mosconi

**Affiliations:** 1 Pathology Unit, Department of Experimental, Diagnostic and Specialty Medicine, Alma Mater Studiorum-University of Bologna, Bologna, Italy; 2 Diagnostic and Interventional Radiology Unit, Department of Diagnostic and Preventive Medicine, Via Albertoni 15, 40136 Bologna, Italy; 3 Unit of Endocrinology and Diabetes Prevention and Care, Department of Medical and Surgical Sciences, University of Bologna, Bologna, Italy; 4 Division of Pancreatic Surgery, Department of Internal Medicine and Surgery (DIMEC), Alma Mater Studiorum, University of Bologna, Bologna, Italy; 5 Nuclear Medicine Unit, Department of Experimental, Diagnostic and Specialty Medicine, Alma Mater Studiorum, University of Bologna, Bologna, Italy; 6 Pathology Unit, Maggiore Hospital, Bologna, Italy

**Keywords:** Texture analysis, Radiomics, Pheocromocytoma, Adrenal, Computed tomography

## Abstract

**Objectives:**

Pheochromocytomas are rare tumors which can present with heterogeneous secretion profiles, clinical manifestations, and radiologic appearance. Under a histopathological point of view, they can be characterized as more or less aggressive with the Pheochromocytoma of the Adrenal gland Scaled Score (PASS) and the Grading system for Adrenal Pheochromocytoma and Paraganglioma (GAPP) score. The aim of this study is to analyze the texture analysis characteristics of pheochromocytoma and identify whether the texture analysis can yield information aiding in the diagnosis and the characterization of those tumors.

**Methods:**

Radiological, biochemical, and histopathological data regarding 30 consecutive patients with histologically confirmed pheochromocytoma were analyzed. Images obtained in the unenhanced, late arterial, venous, and delayed phases were used for the texture analysis.

**Results:**

Urinary epinephrine and metanephrine levels showed a significant correlation (*R*^2^ = 0.946; *R*^2^ = 699) in the multivariate linear model with texture features, as well as Ki-67 (*R*^2^ = 0.397), PASS score (*R*^2^ = 0.182), GAPP score (*R*^2^ = 0.705), and cellularity showed a significant correlation (*R*^2^ = 0.389). The cluster analysis based on radiomic features resulted in 2 clusters, with significative differences in terms of systolic and diastolic blood pressure values at the time of diagnosis (*p* = 0.025), GAPP score (4 vs 6, *p* = 0.05), histological pattern (1–2, *p* = 0.039), and comedonecrosis (0% vs 50%, *p* = 0.013).

**Conclusion:**

In conclusion, our study provides the proof of concept for the use of texture analysis on contrast-enhanced CT images as a noninvasive, quantitative tool for helping in the characterization of the clinical, biochemical, and histopathological features of pheochromocytoma.

## Introduction

Pheochromocytomas (PCCs) are rare tumors that arise from catecholamine-producing chromaffin cells and often are associated with hereditable tumor syndromes [1]. Typical symptoms and signs include headache, tremors, palpitations, sweating, and anxiety. However, up to 25% of the patients do not have signs and symptoms, and up to 30% of PCCs are diagnosed following the discovery of an adrenal incidentaloma (AI) [2].

According to the recent guidelines [1, 3], initial biochemical testing for PCCs should include measurements of plasma free metanephrines or urinary fractionated metanephrines. They recommend that imaging studies to locate PCCs should be initiated once there is clear biochemical evidence of PCCs. They suggest using computed tomography (CT) rather than magnetic resonance imaging (MR) as the first-choice imaging modality because of its excellent spatial resolution for thorax, abdomen, and pelvis.

However, PCCs may present with heterogeneous clinical manifestations and radiologic appearance. Moreover, a few cases may present without elevation in metanephrines (non-secreting) [4].

In addition to that, under a histopathological point of view, PCCs can be characterized as more or less aggressive with the aid of the Pheochromocytoma of the Adrenal gland Scaled Score (PASS) and the recently introduced Grading system for Adrenal Pheochromocytoma and Paraganglioma (GAPP) score [5–7].

For these reasons, it appears relevant for the clinical management to provide the physician not only the diagnosis of PCCs, already a difficult task, but also information relative to the secretion properties and the histopathological aggressive nature [8, 9].

Radiomics, through the extraction of a multitude of date from radiologic images, could be the next step towards a “precision medicine” approach on these tumors [10].

In recent literature, it has been described that a radiomic evaluation of a lesion can predict both the secretion profile and histological characteristics [11–15].

The aim of this study is to study thoroughly the texture analysis characteristics of PCCs and identify whether the texture analysis can yield information aiding in the diagnosis and the characterization of those tumors.

## Methods

### Patients

We retrieved clinical and radiological data from 30 consecutive patients with histologically confirmed PCCs, who have been evaluated at the Endocrinology and Diabetes Prevention and Care Unit of the S. Orsola Policlinic between April 2007 and December 2018. We included patients diagnosed with pheochromocytoma either during the study of resistant hypertension (*n* = 20) or during the hormonal workup of an incidentally discovered adrenal mass (incidentaloma) (*n* = 10). The study conforms to the ethics guidelines of the Declaration of Helsinki, and data collection and analyses had already previously been approved by our Institutional Review Board. All patients provided informed written consent for the processing of personal data according to The Italian Data Protection Authority law (legislative decree no. 196 of 2003 and no. 101 of 2018).

### Clinical and hormonal data

Clinical and hormonal data were retrieved at the time of diagnosis, before adrenalectomy, and analyzed retrospectively. Diagnosis of pheochromocytoma was done according to the current guidelines [1]. Briefly, all patients underwent measurement of 24-h urine metanephrines, after having withdrew medication potentially interfering with the hormonal assays for at least 4 weeks and avoided catecholamine-containing foods for at least 7 days before urine collection.

After adrenalectomy, patients underwent periodical follow-up (mean follow-up was 5.3 years). Metastases were identified in 3/30 patients (1 month, 4 and 12 years after adrenalectomy).

### Computed tomography images

All patients except five underwent a tri-phase contrast-enhanced computed tomography (CECT) imaging; Patients who did not undergo the tri-phase CECT lacked the arterial phase. Tri-phase CECT included an unenhanced phase, an arterial phase, and a venous phase performed during the same examination. All the examinations were conducted with a 64-MDCT VCT Light-Speed CT scanner (from GE Healthcare) with 2 mm slice thickness.

After infusion of the contrast agent at a rate of 3–4 mL/s, the arterial phase was determined using a region of interest (ROI) placement in the abdominal aorta at the level of the celiac artery and the CT scan was initiated when achieving a peak of at least 150 Hounsfield units (HU) within the ROI; the late arterial phase, venous phase (70–80 s), and a late phase (15 min) were then acquired. For all the protocols, 100–150 mL (based on the patient’s weight) of 350 mg I/mL low-osmolar contrast agent (Iomeron 350, Bracco, Italy) was administered intravenously.

### Image processing

**Fig. 1:**
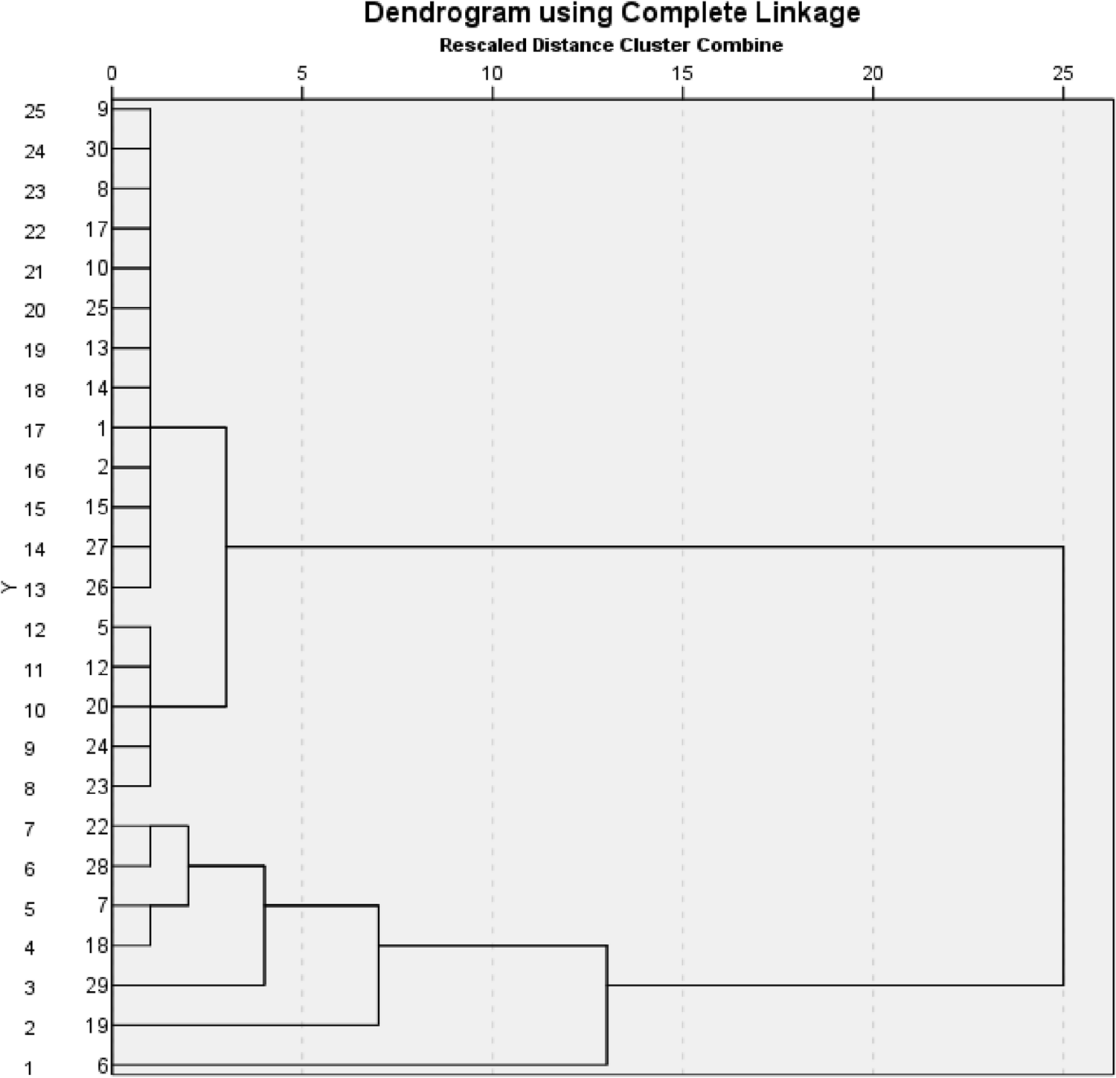
Dendrogram showing the cluster analysis. On the x-axis, the distance between cluster is reported, while on the y-axis, database entries are shown

Images obtained in the unenhanced, late arterial, venous, and delayed phases were used for the texture analysis. For each patient, a volume of interest (VOI) of the whole lesion for each phase was selected by means of manual segmentation (Fig. 1) by one radiologist with 15 years of experience in adrenal imaging (C.M.). The feature extraction was carried out using the LIFEx software [16]. Both first-order and second-order features were analyzed. First-order features are extracted evaluating the mean, skewness, and kurtosis of the distribution of Hounsfield Units (HU) of the voxels in the VOI, as well as geometrical features of the lesions such as compacity or sphericity of the VOI. Second-order features are derived from matrixes, calculating, for each voxel in pair with the adjacent ones, indices such as homogeneity, contrast, correlation, entropy, and dissimilarity for the grey-level co-occurrence matrix (GLCM) [16, 17]. This approach was purely explorative and not prognostic; thus, no derivation and validation cohorts were planned.

### Histopathology

Pheochromocytoma was confirmed at histopathology in all cases.

Formalin-fixed paraffin-embedded tissue blocks from PCC tumors underwent formal histopathologic analysis. Hematoxylin and eosin staining and immunohistochemistry (IHC) were performed on selected tumor blocks.

All hematoxylin and eosin-stained slides of PCC specimens were reviewed by two experienced endocrine pathologists based upon PASS score [7] and GAPP scoring system classification [5]. PASS was categorized as < 4 or ≥ 4, consistent with the established cut-point for risk of metastatic disease. GAPP score was categorized as 0–2: well differentiated; 3–6: moderately differentiated; and 7–10: poorly differentiated, based on published cut-points for risk stratification.

IHC staining for Ki-67 was performed using an automated IHC staining instrument (Benchmark; Ventana Medical Systems, Tucson, USA), the UltraView Universal DAB kit (Ventana Medical Systems), and a Ki-67 antibody (clone 30-9 Ventana, USA).

The evaluation of the proliferative index (Ki-67) in the neoplastic population was carried out quantitatively using image analysis with the Image-Pro Plus 5.1 software (Media Cybernetics Inc., Silver Spring, MD, USA) in the most proliferative foci in at least forty ×200 magnification fields, and expressed as the ratio (%) between the positive neoplastic cells and the total neoplastic cells. The number of Ki-67-positive cells per 100 PCC cells was designated as the labeling index in the hottest spot.

IHC staining for SDHB was also performed using an automated IHC staining instrument (Benchmark; Ventana Medical Systems), the UltraView Universal DAB kit (Ventana Medical Systems), and an SDHB antibody (rabbit polyclonal HPA002868, 1:400 dilution; Sigma—Aldrich, St Louis, MO, USA). Cases with any definite granular cytoplasmic staining (mitochondrial pattern) were scored as positive. The proportion of positive granular cytoplasmic staining varied greatly between positive cases. Weak diffuse cytoplasmic staining was occasionally and heterogeneously observed in combination with definite granular cytoplasmic staining, which was scored as ‘positive’. Cases with completely absent staining or only weak diffuse cytoplasmic staining, in contrast to the positive internal controls (endothelial cells, sustentacular cells, and lymphocytes), were scored as ‘negative’.

### Statistical analysis

The categorical variables were reported as number of cases and percentages and were compared using the Chi-squared test or the Fisher’s exact test when appropriate. The continuous variables were reported as means and standard deviations, and compared between the subgroups using the Mann–Whitney *U* test. The continuous variables with non-normal distribution, determined as such by the mean of the Kolmogorov–Smirnov’s test, were normalized using logarithms. Linear and logistic regression models were used to evaluate the correlation between radiomic features, histological, and clinical data; where no data were available regarding the arterial features of the 5 patients without it, they have been excluded from the analysis. The forward stepwise likelihood ratio method was used as method of selection for the variables of the multivariate models. A cluster analysis on the 25 patients with complete linkage (farthest neighbor) was performed to determine the existence and the relevance of a “radiomic signature”. Two tailed *p* values < 0.05 were considered statistically significant. The analyses were carried out using IBM SPSS 27.0 (Armonk, NY: IBM Corp).

## Results

### Clinical and laboratory data correlations

The clinical and hormonal characteristics of the patients are presented in Table 1, whereas the results of the multivariate linear model to assess the association between hormonal and radiological data are expressed in Table 2.

**Table 1:** Descriptive statistics

1.A—clinical characteristics		
	Mean	Std. Dev.
Height (cm)	163.9	8.3
Weight (kg)	70.8	13.7
Body mass index	26.3	4.1
Systolic pressure	136.3	22.0
Diastolic pressure	84.5	13.9
Heart rate	70.7	8.3
Presence of symptoms, *n* (%)	16	(51.6%)
Max. diameter (mm)	33.7	13.6
Min. diameter (mm)	28.3	12.0

1.B—histopathological characteristics		
	Mean	Std. Dev.
KI-67	2.4	1.7
PASS	5.0	3.1
GAPP	4.0	2.1
SDHB	2.2	1.1
Histological pattern	0.9	0.9
Cellularity	0.9	0.8
Vascular/capsular invasion, *n* (%)	16	(51.6%)
Comedonecrosis, *n* (%)	4	(12.9%)
Metastasis/relapse, *n* (%)	3	(9.7%)

1.C—hormonal characteristics			
	Ref.	Mean	Std. Dev.
Urinary epinephrine (µg/creatinine g)	1–44	32.7	45.1
Urinary norepinephrine (µg/creatinine g)	9–112	342.1	603.5
Urinary dopamine (µg/creatinine g)	30–350	562.6	1247.0
Vanillylmandelic acid (mg/creatinine g)	< 6	12.9	18.9
Homovanillic acid (mg/creatinine g)	< 7	5.5	5.0
Urinary metanephrine (µg/creatinine g)	27–200	1028.9	1532.1
Urinary normetanephrine (µg/creatinine g)	46–400	1785.5	1918.1

**Table 2:** Linear and binary logistic multivariate models

Parameters	aconvmax	aconvmean	aconvmin	aglcmen	ahistoku	ahistosk	ashapecom	ashapevolvx	pconvmax	pconvmin	pconvstd	pglcmcontr	pglcmen	phistoku
Urinary epinephrine	0.658		− 0.76	9231	– 29.8		– 8.29		0.284					
*P *value	< 0.001		< 0.001	< 0.001	< 0.001		0		< 0.001					
Urinary norepinephrine	0.007									– 0.01				
*P *value	< 0.001									0.02				
Urinary metanephrine		– 0.01				0.905								0.337
*P *value		0.030				< 0.001								0.02
Urinary normetanephrine	31.6			97,031										
*P *value	< 0.001			0.01										
KI-67											0.155			
*P *value											0			
PASS TOT														
*P *value														
GAPP TOT	0.020			112.3				2E-05						– 0.829
*P *value	0			0.01				0.01						0.02
SDHB														0.453
*P *value														0.03
SDHB (0–2 vs 3)										– 0.05				
*P *value										0.1				
Histological pattern														
*P *value														
Histological pattern (0 vs 1–2)														
*P *value														
Cellularity														
*P *value														
Vanillylmandelic acid												0.062		
*P *value												0.01		
Homovanillic acid														
*P *value														
Vascular/capsular invasion														
*P *value														
Presence of symptoms													153.3	
*P *value													0.190	

Parameters	pshapecom	pshapevolvx	vconvmean	vconvmin	vconvstd	vglcmcontr	vglcmcorr	vglcmen	vhistosk	vshapecom	vshapevolml	Constant	*R*^2^
Urinary epinephrine					3.431							– 236.6	0.946
*P *value					0.02								
Urinary norepinephrine												0.6	0.574
*P *value													
Urinary metanephrine												2.1	0.699
*P *value													
Urinary normetanephrine												– 6035	0.690
*P *value													
KI-67									– 1.118			– 2.9	0.397
*P *value									0.05				
PASS TOT		5E-05										4.1	0.182
*P *value		0.04											
GAPP TOT												0.7	0.705
*P *value													
SDHB	3.856											– 2.1	0.521
*P *value	0												
SDHB (0–2 vs 3)			0.467									– 16.1	
*P *value			0.03										
Histological pattern										0.382		– 0.4	0.361
*P *value										0			
Histological pattern (0 vs 1–2)										2.164		– 6.7	
*P *value										0.03			
Cellularity					– 0.13							3.3	0.389
*P *value					0								
Vanillylmandelic acid								20.2				− 0.4	
*P *value								< 0.001					
Homovanillic acid				– 0.19								− 9.1	
*P *value				0.01									
Vascular/capsular invasion						– 0.78						3.4	
*P *value						0.02							
Presence of symptoms							– 36.0	123.2			0.247		
*P *value							0.03	0.08			0.040		

Urinary metanephrine levels showed a significant correlation (*R*^2^ = 699) in the multivariate linear model with the skewness (*B* = 0.905, *p* < 0.001), the kurtosis (*B* = 0.337, *p* = 0.015), and the mean value (*B* =  − 0 to 007, *p* = 0.030) of the distribution of HU in the arterial phase, whereas urinary normetanephrine levels showed a significant correlation (*R*^2^ = 0.690) in the multivariate linear model with the maximum value of HU (*B* = 31.649, *p* < 0.001) and the energy (*B* = 97030.659, *p* = 0.007) calculated in the arterial phase.

Urinary epinephrine levels showed a significant correlation (*R*^2^ = 0.946) with energy (*B* = 9230.862, *p* < 0.001), and the distribution of HU in terms of minimum (*B* =  − 0.759, *p* < 0.001), maximum (*B* = 0.658, *p* < 0.001), and kurtosis (*B* =  − 29.753, *p* < 0.001) derived from the arterial phase, the maximum value of HU detected in the portal phase (*B* = 0.284, *p* < 0.001), the standard deviation of the distribution of HU in the unenhanced phase (*B* = 3.431, *p* = 0.016), and the shape compacity (*B* = − 8.292, *p* = 0.001). Urinary norepinephrine levels showed a significant correlation (*R*^2^ = 0.574) with the maximum HU value detected in the arterial phase (*B* = 0.007, *p* < 0.001) and the minimum value in the portal phase (*B* =  − 0.006, *p* = 0.022).

### Histopathological characteristics

The histopathological characteristics of the tumors are reported in Table 3. As shown in Table 4, the multivariate linear model analysis highlighted that Ki-67 had a significant correlation (*R*^2^ = 0.397) with the standard deviation of the HU values distribution in the portal phase (*B* = 0.155, *p* = 0.001) and the skewness of the distribution in the unenhanced phase (*B* =  − 1.118, *p* = 0.047). PASS score showed a significant, yet weak, correlation (*R*^2^ = 0.182) in the multivariate linear model with the volume of the lesion (*B* = 5.33 × 10^− 5^, *p* = 0.037). GAPP score showed a significant correlation (*R*^2^ = 0.705) in the multivariate linear model with the Energy (*B* = 112.272, *p* = 0.006) and maximum HU value (*B* = 0.002, *p* = 0.003) in the arterial phase, kurtosis in the distribution of HU in the portal phase (*B* =  − 0.829, *p* = 0.023), and volume of the lesion (*B* = 2.25 × 10^− 5^, *p* = 0.008).

Tissue expression of SDHB showed a significant correlation (*R*^2^ = 0.521) with the kurtosis of the distribution of HU in the portal phase (*B* = 0.453, *p* = 0.031), and the shape compacity (*B* = 3.856, *p* = 0.001).

Histological pattern showed a significant correlation (*R*^2^ = 0.361) in the multivariate linear model with the compacity of the lesion (*B* = 0.382, *p* = 0.003).

Cellularity showed a significant correlation (*R*^2^ = 0.389) in the multivariate linear model with the standard deviation of the HU in the distribution calculated in the unenhanced phase (*B* = − 0.128, *p* = 0.002).

### Cluster analysis

The cluster analysis based on radiomic features was performed on 25 patients, because 5 had incomplete clinical data; the analysis identified 2 clusters, with 18 patients in cluster 1 and 7 patients in cluster 2. The dendrogram of the cluster analysis is shown in Fig. 1.

The radiomic, clinical, and histopathological characteristics of the two clusters are shown in Table 3. When compared to cluster 2, patients belonging to cluster 1 showed higher compacity (15.4 vs 8.9, *p* = 0.001), lower volume (10.2 mL vs 42.7 mL, *p* < 0.001), lower maximum HU value detected in the unenhanced (86.1 vs 94.7, *p* = 0.040) and in the arterial (197.4 vs 241.9, *p* = 0.021) phase, and higher minimum HU value in the portal phase (− 24.2 vs − 53.3, *p* = 0.046). Patients belonging to cluster 2 had higher systolic and diastolic blood pressure values at the time of diagnosis (*p* = 0.025), and higher GAPP score (4 vs 6, *p* = 0.05), histological pattern (1–2, *p* = 0.039), and comedonecrosis (0% vs 50%, *p* = 0.013) at histopathology, than subjects in cluster 1. Even if it did not reach the significance threshold, it is worth noting that all patients with metastatic disease were included in cluster 2 (0–28.6%, *p* = 0.07).

**Table 3:** Clusters comparison

	Cluster 1 (*n* = 18)	Cluster 2 (*n* = 7)	*P* value
Systolic pressure	129.1	154.3	0.025
Diastolic pressure	81.8	93.6	0.050
Max. diameter (mm)	27.6	49.0	< 0.001
Min. diameter (mm)	22.2	42.0	< 0.001
GAPP	4	6	0.050
Histologic pattern	1	2	0.039
Comedonecrosis, *n*	0	3	0.013
Metastasis/relapse, *n*	0	2	0.070
vconvmax	86.1	94.7	0.040
vshapevolml	10.2	42.7	< 0.001
vshapevolvx	5743	29,894	< 0.001
vshapecom	2.84	4.99	0.004
aconvmax	197	242	0.021
ahistosk	− 0.410	0.170	0.069
ashapevolml	7.1	42.1	< 0.001
ashapevolvx	10,093	81,245	< 0.001
ashapecom	15.4	8.9	0.001
pconvmin	– 24.2	– 53.3	0.046
pshapevolml	10.2	42.2	< 0.001
pshapevolvx	11,915	52,372	0.001
pshapecom	4.20	7.09	0.004

## Discussion

This study evaluated the correlation between laboratory results, clinical characteristics, histopathological findings and features extracted from texture analysis of CT scans in a cohort of pheochromocytoma.

Objectifying findings on CT scans is already a common clinical practice, at least when regarding the calculation of absolute and relative wash-out of a lesion [18]. Texture analysis can help to quantify heterogeneity in density and vascularization to a further level, to an extent that is not accessible to the human eye alone.

As far as the laboratory results are concerned, highly secretive tumors showed an increase in texture features that have in common a general meaning of “uniformity” and “vascularization”, namely higher energy on the GLCM matrix, higher kurtosis, lower minimum and higher maximum value of HU in the contrast phases.

Regarding the histopathological characteristics, indices of rapid growth and higher tumor grade (ki67 and GAPP score) showed a significative correlation with features indicating “heterogeneity” in the unenhanced and portal phase, and “uniformity” in the arterial phase.

Moreover, this study identified two different cluster with common texture features, one characterized by a more symptomatic presentation and aggressive histology and presenting higher HU values in the arterial phase and lower HU values in the venous phase, as well as lower maximum HU value detected in the unenhanced phase.

These results are consistent with previous findings demonstrating higher heterogeneity in malignant tumors of the adrenal glands [19].

The results of our pilot study provide useful information that may be applied into the clinical practice. Indeed, the association between radiomic signature and specific secretory profile provide support to the utility of the texture analysis by CT scan as a potential aid in overcoming the current limitations of the biochemical diagnosis of these tumors, mostly due to several assay interference. In addition, the analysis of the clinical and histopathological characteristics of the two clusters identified by radiomic features further support a potential role of the texture analysis in identifying tumors with different behavior, even though this aspect should be confirmed in larger targeted studies, investigating also the genetic background of those tumors.

Oftentimes radiomics suffers from the “black box” problem, especially when presenting higher order features in complex machine learning approaches, providing further difficulties in the interpretation of the data. On the contrary, most of the results presented in this work are easily deployable through most PACS software, as the measurement of mean value and standard deviation is commonly implemented and the standard deviation could be easily integrated and more available in the near future.

The main limitation of the study is the small cohort, which hinders the robustness of the presented models, and impedes a validation process. However, it must be considered that pheochromocytomas are rare tumors.

Furthermore, another limit is that this study does not address the main radiologic diagnostic challenge regarding the adrenal glands, which is discriminating malignant from benign lesions. This is a potential use of texture analysis, which already demonstrated an accuracy up to 93% in this task [20]. However, this study aimed to give even more purpose to the data extracted in the setting of the studies regarding the discrimination of adrenal incidentalomas. Moreover, the demonstrated link between radiomic and histopathologic feature could help identify patient at higher risk of metastases, once and endocrinological diagnosis of PCC is obtained, with potential management implications, if confirmed by larger and prospective trials.

Another future development of this work could be investigating how experienced radiologist could express qualitatively on the basis of the presented results, this could serve to verify the previous statements, both that of the immediate semiotic correlation regarding the heterogenicity of the tumor, and the immediate clinical applicability of the results.

In conclusion, our study provides the proof of concept for the use of texture analysis on contrast-enhanced CT images as a noninvasive, quantitative tool for helping in the characterization of the clinical, biochemical and histopathological features of pheochromocytoma. A few features were identified as excellent discriminators of lesions, which may overcome the current limitations in biochemical testing and obviate the need for additional imaging. Future work may entail training of machine learning algorithms to be able to differentiate tumors. This would move the technique one step closer to clinical application.

## Abbreviations

PCC: Pheochromocytoma
AI: Adrenal incidentaloma
CT: Computed tomography
MR: Magnetic resonance
PASS: Pheochromocytoma of the adrenal gland scaled score
GAPP: Grading system for adrenal pheochromocytoma and paraganglioma
VOI: Volume of interest
HU: Hounsfield unit
GLCM: Grey-level co-occurrence matrix
IHC: Immunohistochemistry
SDHB: Succinate dehydrogenase subunit B
HPF: High-powered field
MPF: Medium-powered field (magnification at ×20 with objective lens and 10 × ocular lens)
